# Supplementary material for: Eco-Efficiency Analysis for the Russian Cities along the Northern Sea Route: A Data Envelopment Analysis Approach Using an Epsilon-Based Measure Model
Source: Int J Environ Res Public Health. 2021 Jun 5;18(11):6097. doi: 10.3390/ijerph18116097 (PMC8201002; doi:10.3390/ijerph18116097)
Supplement: Supplementary file 1 [file ijerph-18-06097-s001.zip › ijerph-1229085-supplementary.pdf]

Supplementary Materials for

Eco-efficiency analysis for the Russian cities along the Northern Sea Route: a data envelopment analysis approach using an epsilon-based measure model

Shuaiyu Yao, Mengmeng Chen, Dmitri Muravev, Wendi Ouyang

Correspondence to: mmchen_hcgufe@hotmail.com

This file includes:

Tables S1, S2, S3, S4, S5, S6, S7, S8, S9, and S10

Table S1. The input and output data of the Russian cities along the Northern Sea Route in 2010

No.	Russian city	Population (million people)	Capital (billion US dollars)	Public investment (billion US dollars)	Water supply (average daily consumption in thousand m^3)	Energy supply (specific fuel consumption for electric power generation by thermal power plants in grams of conventional fuel/kWh)	GRP (million US dollars)	GHG (thousand tons of CO_2 equivalent)	Solid waste (thousand tons)	Water pollution (million m^3)

1	St. Petersburg	4.8790	15.4500	5.1686	1994.7000	284.1000	25632.2200	234.2000	1635.0000	1105.0000
2	Murmansk	0.3070	0.6500	0.0120	398.9400	323.5400	2007.3360	3.3200	90.6000	461.0000
3	Kandalaksha	0.0350	0.0080	0.0001	63.8304	51.7664	143.2200	0.6308	19.9320	82.9800
4	Onega	0.0210	0.0027	0.0006	6.9012	5.7476	93.0930	2.9092	2.4234	10.5560
5	Arkangelsk	0.3550	0.1914	0.0457	492.9400	410.5400	1201.2647	207.8000	173.1000	754.0000
6	Naryan Mar	0.0217	1.1214	0.3826	44.6813	36.2365	1149.7143	2.4236	66.1380	336.5300
7	Dudinka	0.0222	0.0270	0.0060	319.1520	258.8320	55.1100	0.5046	15.9456	66.3840
8	Providenya	0.0020	0.0000	0.0060	5.7012	4.5476	24.3291	0.4308	10.5020	74.4600
9	P.-Kamchatskiy	0.1798	0.3643	0.1114	300.0029	230.3605	795.7143	20045.0000	77.0100	382.6300
10	Vanino	0.0170	0.0014	0.0389	52.3409	42.4484	76.9153	0.5362	19.9320	82.9800
11	Vladivostok	0.5920	1.1000	0.0078	443.2667	271.7736	1713.2143	26.0222	181.6667	122.7778
12	Hakhodka	0.1597	2.8700	0.0410	320.4110	348.9590	894.3400	99.7440	83.0880	361.9200
13	Novodvinsk	0.0406	0.0800	0.0008	58.0857	47.1074	154.5238	3.1507	111.7732	437.4890
14	Vorkuta	0.0705	0.0500	0.0150	78.4156	63.5950	61.6530	2.8104	19.6200	13.2600
15	Salekhard	0.0425	0.0900	0.0405	54.8910	54.0558	1988.2200	1.9673	16.6770	13.2600
16	Nadym	0.0466	0.0001	0.0000	46.6573	45.9474	1811.2300	1.6722	14.1755	11.2710
17	N. Urengoy	0.1041	0.1667	0.0182	23.3200	293.1256	4321.4500	84.7824	70.6248	307.6320
18	Noyabrsk	0.1106	2.5600	1.6640	20.7548	255.0192	3761.4500	75.4563	61.4436	264.5635
19	Norilsk	0.1754	0.2786	0.1671	362.0644	394.3237	817.7065	112.7107	93.8894	408.9696
20	Monchegorsk	0.0454	0.0650	0.0585	49.4019	48.6502	192.8745	13.5652	28.2499	123.0528
21	Apatity	0.0597	0.0170	0.0145	61.7523	60.8127	312.8745	21.1956	35.3124	153.8160
22	Kirovsk	0.0286	0.1100	0.0990	38.4237	37.8390	166.4180	7.0675	14.7182	64.1105
23	Revda	0.0619	0.0260	0.0099	68.0648	67.0292	205.0979	2.4394	20.6795	16.4424
24	Olenegorsk	0.7954	0.0290	0.0033	527.4873	323.4106	2681.7607	30.9664	216.1833	146.1056
25	Kovdor	0.0188	0.0376	0.0245	7.4717	192.0192	70.9708	45.2738	36.8661	158.7381
26	Kola	0.0104	0.0226	0.0147	4.4830	115.2115	48.6101	27.1643	22.1197	95.2429

27	Nikel	0.0128	0.0282	0.0184	5.6038	144.0144	12.1525	33.9554	27.6496	119.0536
28	Bilibino	0.0055	0.0184	0.0119	3.6425	93.6094	58.0587	22.0710	17.9722	77.3848

Source: Official Statistics of Russian Federal State Statistic Service [52]

Table S2. The input and output data of the Russian cities along the Northern Sea Route in 2011

No.	Russian city	Population (million people)	Capital (billion US dollars)	Public investment (billion US dollars)	Water supply (average daily consumption in thousand m^3)	Energy supply (specific fuel consumption for electric power generation by thermal power plants in grams of conventional	GRP (million US dollars)	GHG (thousand tons of CO_2 equivalent)	Solid waste (thousand tons)	Water pollution (million m^3)
1	St. Petersburg	4.8990	16.3100	5.4771	1922,9	283.9000	27391.4286	221.6000	1682.0000	1099.0000
2	Murmansk	0.3070	0.7600	0.0230	383.3300	319.2100	2132.7000	3.8400	107.2000	439.0000
3	Kandalaksha	0.0350	0.0080	0.0002	61.3328	51.0736	168.3200	0.7296	23.5840	79.0200
4	Onega	0.0210	0.0046	0.0025	6.6826	5.6869	109.4080	2.8448	3.1178	9.4080
5	Arkangelsk	0.3550	0.3314	0.1771	477.3300	406.2100	1367.3072	203.2000	222.7000	723.0000
6	Naryan Mar	0.0213	1.1666	0.1733	42.9330	35.7515	1207.4286	2.8032	78.2560	320.4700
7	Dudinka	0.0222	0.0280	0.0070	306.6640	255.3680	61.2300	0.5837	18.8672	63.2160
8	Providenya	0.0020	0.0000	0.0070	5.4826	4.4869	25.6256	0.5296	14.1540	70.5000
9	P.-Kamchatskiy	0.1795	0.3957	0.1331	288.2642	227.2775	834.2857	18734.0000	91.1200	364.3700
10	Vanino	0.0170	0.0015	0.0411	50.2929	41.8804	81.5643	0.6202	23.5840	79.0200
11	Vladivostok	0.5921	1.2600	0.0150	468.2210	268.1364	2528.5000	24.6222	186.8889	122.1111

12	Hakhodka	0.1595	2.5600	0.0366	310.2645	345.2785	784.4300	97.5360	106.8960	347.0400
13	Novodvinsk	0.0406	0.0900	0.0009	55.8128	46.4770	196.3333	3.6442	132.2526	416.6110
14	Vorkuta	0.0705	0.0700	0.0150	75.3473	62.7439	59.8700	2.6592	20.1840	13.1880
15	Salekhard	0.0427	0.1500	0.0675	52.7431	53.3323	2288.3300	1.8614	17.1564	13.1880
16	Nadym	0.0467	0.0003	0.0002	44.8317	45.3325	2156.3400	1.5822	14.5829	11.2098
17	N. Urengoy	0.1055	0.1887	0.0304	23.6246	290.0339	4843.2400	82.9056	90.8616	294.9840
18	Noyabrsk	0.1105	2.9900	1.9435	21.0259	252.3295	4283.2400	73.7860	79.0496	253.6862
19	Norilsk	0.1753	0.4714	0.2829	350.5989	390.1647	931.1391	110.2157	120.7925	392.1552
20	Monchegorsk	0.0453	0.0720	0.0648	47.4688	47.9991	228.4500	13.2649	36.3446	117.9936
21	Apatity	0.0597	0.0210	0.0179	59.3360	59.9989	348.4500	20.7264	45.4308	147.4920
22	Kirovsk	0.0286	0.1400	0.1260	36.9202	37.3326	171.4530	6.9110	18.9356	61.4747
23	Revda	0.0619	0.0280	0.0106	65.4015	66.1321	224.8155	2.3082	21.2739	16.3531
24	Olenegorsk	0.7941	0.0400	0.0040	490.0949	315.7536	4793.5502	28.4674	227.8189	143.9900
25	Kovdor	0.0187	0.0440	0.0286	7.5693	189.3295	80.8158	44.2716	47.4298	152.2117
26	Kola	0.0104	0.0264	0.0171	4.5416	113.5977	55.3533	26.5630	28.4579	91.3270
27	Nikel	0.0128	0.0330	0.0214	5.6770	141.9971	13.8383	33.2037	35.5723	114.1588
28	Bilibino	0.0055	0.0214	0.0139	3.6900	92.2981	66.1126	21.5824	23.1220	74.2032

Source: Official Statistics of Russian Federal State Statistic Service [52]

Table S3. The input and output data of the Russian cities along the Northern Sea Route in 2012

No.	Russian city	Population (million people)	Capital (billion US dollars)	Public investment (billion US dollars)	Water supply (average daily consumption in thousand m^3)	Energy supply (specific fuel consumption for electric power generation by thermal power plants in grams of conventional fuel/kWh)	GRP (million US dollars)	GHG (thousand tons of CO_2 equivalent)	Solid waste (thousand tons)	Water pollution (million m^3)
1	St. Petersburg	4.9530	17.6614	5.0314	1853.3000	283.7000	30541.4286	215.3000	1723.0000	1089.0000
2	Murmansk	0.3050	0.8543	0.1700	370.6600	315.8800	2407.4640	4.1700	123.5000	417.0000
3	Kandalaksha	0.0340	0.0092	0.0003	59.3056	50.5408	189.3400	0.7923	27.1700	75.0600
4	Onega	0.0210	0.0039	0.0020	6.5052	5.6403	123.0710	2.4990	3.7982	9.9540
5	Arkangelsk	0.3560	0.2757	0.1400	464.6600	402.8800	1699.0536	178.5000	271.3000	711.0000
6	Naryan Mar	0.0224	1.2221	0.6293	41.5139	35.3786	1326.8571	3.0441	90.1550	304.4100
7	Dudinka	0.0224	0.0310	0.0080	296.5280	252.7040	73.2100	0.6338	21.7360	60.0480
8	Providenya	0.0020	0.0000	0.0080	5.3052	4.4403	27.7888	0.5923	17.7400	66.5400
9	P.-Kamchatskiy	0.1798	0.4186	0.1204	278.7363	224.9066	847.1429	17982.0000	104.9750	346.1100
10	Vanino	0.0167	0.0025	0.0709	48.6306	41.4435	89.3214	0.6735	27.1700	75.0600
11	Vladivostok	0.5975	1.3500	0.1105	411.8444	265.3392	4086.0114	23.9222	191.4444	121.0000
12	Hakhodka	0.1589	2.7300	0.0390	302.0290	342.4480	753.3300	85.6800	130.2240	341.2800
13	Novodvinsk	0.0403	0.1000	0.0001	53.9681	45.9921	276.2057	3.9573	152.3620	395.7330
14	Vorkuta	0.0671	0.0800	0.0230	72.8569	62.0894	79.6000	2.5836	20.6760	13.0680
15	Salekhard	0.0443	0.1900	0.0855	50.9999	52.7760	2432.4500	1.8085	17.5746	13.0680
16	Nadym	0.0474	0.0017	0.0010	43.3499	44.8596	2434.7600	1.5372	14.9384	11.1078

17	N. Urengoy	0.1122	0.2046	0.0385	25.1310	287.6563	5289.3300	72.8280	110.6904	290.0880
18	Noyabrsk	0.1092	3.1200	2.0280	22.3666	250.2610	4729.3300	64.8169	96.3006	249.4757
19	Norilsk	0.1773	0.7000	0.4200	341.2928	386.9662	1028.1152	96.8184	147.1531	385.6464
20	Monchegorsk	0.0446	0.0900	0.0810	45.8999	47.4984	263.2300	11.6525	44.2762	116.0352
21	Apatity	0.0592	0.0250	0.0213	57.3748	59.3730	383.2300	18.2070	55.3452	145.0440
22	Kirovsk	0.0283	0.1610	0.1449	35.6999	36.9432	179.4420	6.0709	23.0679	60.4543
23	Revda	0.0618	0.0320	0.0122	63.2398	65.4422	255.3443	2.2426	21.7925	16.2043
24	Olenegorsk	0.7879	0.0400	0.0040	490.0949	315.7536	4793.5502	28.4674	227.8189	143.9900
25	Kovdor	0.0184	0.0459	0.0298	8.0520	187.2610	89.2326	38.8902	57.7804	149.6854
26	Kola	0.0103	0.0275	0.0179	4.8312	112.3566	61.1182	23.3341	34.6682	89.8112
27	Nikel	0.0126	0.0344	0.0224	6.0390	140.4457	15.2796	29.1676	43.3353	112.2641
28	Bilibino	0.0055	0.0224	0.0145	3.9253	91.2897	72.9981	18.9589	28.1679	72.9716

Source: Official Statistics of Russian Federal State Statistic Service [52]

Table S4. The input and output data of the Russian cities along the Northern Sea Route in 2013

No.	Russian city	Population (million people)	Capital (billion US dollars)	Public investment (billion US dollars)	Water supply (average daily consumption in thousand m^3)	Energy supply (specific fuel consumption for electric power generation by thermal power plants in grams of conventional fuel/kWh)	GRP (million US dollars)	GHG (thousand tons of CO_2 equivalent)	Solid waste (thousand tons)	Water pollution (million m^3)
1	St. Petersburg	5.0280	19.8471	6.7871	1808.8000	275.2000	32821.1100	211.2000	1758.0000	1071.0000

2	Murmansk	0.3020	1.0016	0.2100	361.7600	314.2200	2802.2134	4.6500	142.2000	395.0000
3	Kandalaksha	0.0350	0.0114	0.0007	57.8816	50.2752	210.2200	0.8835	31.2840	71.1000
4	Onega	0.0200	0.0028	0.0011	6.3806	5.6171	136.6430	2.4178	4.4996	9.4080
5	Arkangelsk	0.3580	0.2029	0.0757	455.7600	401.2200	1973.2253	172.7000	321.4000	672.0000
6	Naryan Mar	0.0229	1.3064	3.0800	40.5171	35.1926	1421.7143	3.3945	103.8060	288.3500
7	Dudinka	0.0223	0.0320	0.0080	289.4080	251.3760	79.3300	0.7068	25.0272	56.8800
8	Providenya	0.0020	0.0000	0.0090	5.1806	4.4171	30.2720	0.6835	21.8540	62.5800
9	P.-Kamchatskiy	0.1816	0.4671	0.1303	272.0435	223.7246	904.2857	13532.0000	120.8700	327.8500
10	Vanino	0.0165	0.0020	0.0560	47.4629	41.2257	98.2260	0.7510	31.2840	71.1000
11	Vladivostok	0.6004	1.4100	0.1365	401.9556	263.9448	4831.3571	23.4667	195.3333	119.0000
12	Hakhodka	0.1584	2.9300	0.0419	296.2440	341.0370	772.2300	82.8960	154.2720	322.5600
13	Novodvinsk	0.0399	0.1100	0.0001	52.6723	45.7504	314.4286	4.4129	175.4321	374.8550
14	Vorkuta	0.0644	0.1100	0.0300	71.1075	61.7631	100.6922	2.5344	21.0960	12.8520
15	Salekhard	0.0467	0.2100	0.0945	49.7753	52.4986	2693.8400	1.7741	17.9316	12.8520
16	Nadym	0.0468	0.0014	0.0008	42.3090	44.6238	2732.3400	1.5080	15.2419	10.9242
17	N. Urengoy	0.1165	0.2331	0.0425	26.0848	286.4711	5700.5400	70.4616	131.1312	274.1760
18	Noyabrsk	0.1081	3.7500	2.4375	23.2155	249.2298	5140.5400	62.7108	114.0841	235.7914
19	Norilsk	0.1777	0.6157	0.3694	334.7557	385.3718	1117.5087	93.6725	174.3274	364.4928
20	Monchegorsk	0.0440	0.1100	0.0990	44.7978	47.2488	296.1100	11.2739	52.4525	109.6704
21	Apatity	0.0587	0.0280	0.0238	55.9972	59.0609	416.1100	17.6154	65.5656	137.0880
22	Kirovsk	0.0281	0.1230	0.1107	34.8427	36.7490	174.3330	5.8737	27.3277	57.1383
23	Revda	0.0618	0.0300	0.0114	61.7213	65.0983	265.0587	2.1999	22.2352	15.9365
24	Olenegorsk	0.7804	0.0700	0.0038	478.3271	314.0943	5456.9079	27.9253	232.4467	141.6100
25	Kovdor	0.0180	0.0551	0.0358	8.3576	186.2298	96.9913	37.6265	68.4505	141.4748
26	Kola	0.0101	0.0331	0.0215	5.0145	111.7379	66.4324	22.5759	41.0703	84.8849
27	Nikel	0.0124	0.0414	0.0269	6.2682	139.6724	16.6081	28.2199	51.3379	106.1061

28	Bilibino	0.0055	0.0269	0.0175	4.0743	90.7870	79.3452	18.3429	33.3696	68.9690

Source: Official Statistics of Russian Federal State Statistic Service [52]

Table S5. The input and output data of the Russian cities along the Northern Sea Route in 2014

No.	Russian city	Population (million people)	Capital (billion US dollars)	Public investment (billion US dollars)	Water supply (average daily consumption in thousand m^3)	Energy supply (specific fuel consumption for electric power generation by thermal power plants in grams of conventional fuel/kWh)	GRP (million US dollars)	GHG (thousand tons of CO_2 equivalent)	Solid waste (thousand tons)	Water pollution (million m^3)
1	St. Petersburg	5.1310	19.8471	6.7871	1808.8000	275.2000	32821.1100	211.2000	1758.0000	1071.0000
2	Murmansk	0.2990	1.1871	0.2800	342.5400	311.3100	3048.7329	5.0700	156.3000	376.0000
3	Kandalaksha	0.0340	0.0135	0.0008	54.8064	49.8096	238.4500	0.9633	34.3860	67.6800
4	Onega	0.0200	0.0036	0.0015	6.1116	5.5763	154.9925	2.4668	5.1142	9.2260
5	Arkangelsk	0.3570	0.2586	0.1043	436.5400	398.3100	2146.8161	176.2000	365.3000	659.0000
6	Naryan Mar	0.0234	1.2793	3.7407	38.3645	34.8667	1478.8571	3.7011	114.0990	274.4800
7	Dudinka	0.0224	0.0470	0.0090	274.0320	249.0480	80.0100	0.7706	27.5088	54.1440
8	Providenya	0.0020	0.0000	0.0090	4.9116	4.3763	34.4576	0.7633	24.9560	59.1600
9	P.-Kamchatskiy	0.1827	0.3600	0.1071	257.5901	221.6527	1082.8571	11521.0000	132.8550	312.0800
10	Vanino	0.0161	0.0019	0.0520	44.9412	40.8439	113.2353	0.8188	34.3860	67.6800
11	Vladivostok	0.6032	1.4300	0.1820	380.6000	261.5004	5044.9286	21.7000	199.3333	117.1111
12	Hakhodka	0.1564	3.1500	0.0450	283.7510	338.5635	938.9863	84.5760	175.3440	316.3200

13	Novodvinsk	0.0396	0.0900	0.0009	49.8738	45.3267	325.3810	4.8114	192.8273	356.8240
14	Vorkuta	0.0616	0.1200	0.0400	67.3297	61.1911	112.4989	2.3436	21.5280	12.6480
15	Salekhard	0.0479	0.3400	0.1530	47.1308	52.0124	2944.5500	1.6405	18.2988	12.6480
16	Nadym	0.0458	0.0005	0.0003	40.0611	44.2106	3001.2200	1.3944	15.5540	10.7508
17	N. Urengoy	0.1158	0.3126	0.0689	25.9287	284.3933	6234.4500	71.8896	149.0424	268.8720
18	Noyabrsk	0.1074	4.1100	2.6715	23.0765	247.4222	5674.4500	63.9817	129.6669	231.2299
19	Norilsk	0.1766	0.6400	0.3840	320.6386	382.5768	1233.5761	95.5709	198.1387	357.4416
20	Monchegorsk	0.0435	0.0820	0.0738	42.4177	46.8112	333.2300	11.5023	59.6170	107.5488
21	Apatity	0.0579	0.0290	0.0247	53.0221	58.5140	453.2300	17.9724	74.5212	134.4360
22	Kirovsk	0.0277	0.1430	0.1287	32.9915	36.4087	198.3720	5.9927	31.0604	56.0329
23	Revda	0.0620	0.0310	0.0118	58.4421	64.4954	275.0449	2.0342	22.6905	15.6835
24	Olenegorsk	0.7711	0.0500	0.0039	452.9140	311.1855	5646.9864	25.8230	237.2067	139.3622
25	Kovdor	0.0176	0.0604	0.0393	8.3075	184.4222	107.0651	38.3890	77.8001	138.7380
26	Kola	0.0101	0.0363	0.0236	4.9845	110.6533	73.3323	23.0334	46.6801	83.2428
27	Nikel	0.0121	0.0453	0.0295	6.2307	138.3167	18.3331	28.7918	58.3501	104.0535
28	Bilibino	0.0056	0.0295	0.0192	4.0499	89.9058	87.5862	18.7147	37.9276	67.6348

Source: Official Statistics of Russian Federal State Statistic Service [52]

Table S6. The input and output data of the Russian cities along the Northern Sea Route in 2015

No.	Russian city	Population (million people)	Capital (billion US dollars)	Public investment (billion US dollars)	Water supply (average daily consumption in thousand m^3)	Energy supply (specific fuel consumption for electric power generation by thermal power plants in grams of conventional	GRP (million US dollars)	GHG (thousand tons of CO_2 equivalent)	Solid waste (thousand tons)	Water pollution (million m^3)
1	St. Petersburg	5.1910	14.4057	6.9057	1635.6000	259.6000	43199.6000	182.2000	1831.0000	1032.0000
2	Murmansk	0.3050	1.4429	0.3200	327.1200	307.4500	3432.8966	5.6700	175.2000	363.0000
3	Kandalaksha	0.0320	0.0162	0.0009	52.3392	49.1920	251.9800	1.0773	38.5440	65.3400
4	Onega	0.0200	0.0018	0.0003	5.8957	5.5223	163.7870	2.2120	5.6186	9.0020
5	Arkangelsk	0.3580	0.1300	0.0200	421.1200	394.4500	2417.3313	158.0000	401.3300	643.0000
6	Naryan Mar	0.0239	1.2096	3.1181	36.6374	34.4344	1744.0000	4.1391	127.8960	264.9900
7	Dudinka	0.0222	0.0490	0.0090	261.6960	245.9600	89.7300	0.8618	30.8352	52.2720
8	Providenya	0.0020	0.0000	0.0150	4.6957	4.3223	37.6448	0.8773	29.1140	56.8200
9	P.-Kamchatskiy	0.1810	0.3263	0.0929	245.9942	218.9044	1505.7143	8028.0000	148.9200	301.2900
10	Vanino	0.0158	0.0021	0.0583	42.9181	40.3374	124.6644	0.9157	38.5440	65.3400
11	Vladivostok	0.6046	1.5100	0.2080	363.4667	258.2580	5339.2857	20.2444	203.4444	114.6667
12	Hakhodka	0.1557	2.9400	0.0420	273.7280	335.2825	1246.3173	75.8400	192.6384	308.6400
13	Novodvinsk	0.0392	0.1200	0.0001	47.6287	44.7647	340.4762	5.3808	216.1442	344.4870
14	Vorkuta	0.0604	0.1300	0.0500	64.2987	60.4324	396.8790	2.1864	21.9720	12.3840
15	Salekhard	0.0483	0.1700	0.0765	45.0091	51.3675	3231.5400	1.5305	18.6762	12.3840
16	Nadym	0.0458	0.0023	0.0014	38.2577	43.6624	3200.3400	1.3009	15.8748	10.5264
17	N. Urengoy	0.1151	0.2766	0.0344	25.7806	281.6373	6835.0000	64.4640	163.7426	262.3440

18	Noyabrsk	0.1071	4.3400	2.8210	22.9447	245.0245	6275.0000	57.3730	142.4561	225.6158
19	Norilsk	0.1763	1.1186	0.6711	309.3126	378.8692	1364.1304	85.6992	217.6814	348.7632
20	Monchegorsk	0.0432	0.0800	0.0720	40.5082	46.2308	361.7500	10.3142	65.4971	104.9376
21	Apatity	0.0574	0.0230	0.0196	50.6352	57.7885	481.7500	16.1160	81.8713	131.1720
22	Kirovsk	0.0273	0.1590	0.1431	31.5064	35.9573	195.5630	5.3737	34.1240	54.6725
23	Revda	0.0622	0.0230	0.0087	55.8113	63.6957	338.1086	1.8978	23.1585	15.3562
24	Olenegorsk	0.7663	0.0480	0.0029	432.5253	307.3270	5908.9643	24.0909	242.0989	136.4533
25	Kovdor	0.0174	0.0638	0.0415	8.2601	182.0245	118.3962	34.4238	85.4737	135.3695
26	Kola	0.0099	0.0383	0.0249	4.9561	109.2147	81.0933	20.6543	51.2842	81.2217
27	Nikel	0.0118	0.0479	0.0311	6.1951	136.5183	20.2733	25.8178	64.1052	101.5271
28	Bilibino	0.0056	0.0311	0.0202	4.0268	88.7369	96.8558	16.7816	41.6684	65.9926

Source: Official Statistics of Russian Federal State Statistic Service [52]

Table S7. The input and output data of the Russian cities along the Northern Sea Route in 2016

No.	Russian city	Population (million people)	Capital (billion US dollars)	Public investment (billion US dollars)	Water supply (average daily consumption in thousand m^3)	Energy supply (specific fuel consumption for electric power generation by thermal power plants in grams of conventional	GRP (million US dollars)	GHG (thousand tons of CO_2 equivalent)	Solid waste (thousand tons)	Water pollution (million m^3)
1	St. Petersburg	5.2250	17.3200	9.6900	1603.9000	259.1000	53459.7429	175.8000	1878.0000	1009.0000
2	Murmansk	0.3010	1.7629	0.2700	320.7800	303.3200	3648.2631	6.2300	192.3000	339.0000
3	Kandalaksha	0.0320	0.0183	0.0010	51.3248	48.5312	293.8057	1.1837	42.3060	61.0200

4	Onega	0.0190	0.0026	0.0006	5.8069	5.4645	190.9737	2.2134	6.2062	8.8620
5	Arkangelsk	0.3580	0.1886	0.0414	414.7800	390.3200	2568.9853	158.1000	443.3000	633.0000
6	Naryan Mar	0.0245	1.2157	2.4883	35.9274	33.9718	1809.7143	4.5479	140.3790	247.4700
7	Dudinka	0.0220	0.0540	0.0100	256.6240	242.6560	98.3200	0.9470	33.8448	48.8160
8	Providenya	0.0021	0.0000	0.0170	4.6069	4.2645	41.8286	0.9837	32.8760	52.5000
9	P.-Kamchatskiy	0.1810	0.5327	0.1543	241.2266	215.9638	1815.7143	7923.0000	163.4550	281.3700
10	Vanino	0.0156	0.0019	0.0543	42.0863	39.7956	139.6671	1.0061	42.3060	61.0200
11	Vladivostok	0.6067	1.6400	0.1755	356.4222	254.7888	5617.8571	19.5333	208.6667	112.1111
12	Hakhodka	0.1536	2.4500	0.0350	269.6070	331.7720	1418.6105	75.8880	212.7840	303.8400
13	Novodvinsk	0.0389	0.1400	0.0004	46.7056	44.1634	354.7619	5.9123	237.2405	321.7110
14	Vorkuta	0.0592	0.1100	0.0400	63.0525	59.6206	520.7061	2.1096	22.5360	12.1080
15	Salekhard	0.0485	0.0800	0.0360	44.1368	50.6775	3556.2200	1.4767	19.1556	12.1080
16	Nadym	0.0449	0.0005	0.0003	37.5162	43.0759	3456.8800	1.2552	16.2823	10.2918
17	N. Urengoy	0.1112	0.2331	0.0162	24.9005	278.6885	7489.4300	64.5048	180.8664	258.2640
18	Noyabrsk	0.1066	5.1300	3.3345	22.1615	242.4590	6929.4300	57.4093	157.3538	222.1070
19	Norilsk	0.1774	0.9071	0.5443	304.6559	374.9024	1506.3978	85.7534	240.4459	343.3392
20	Monchegorsk	0.0429	0.0900	0.0810	39.7231	45.6097	393.8190	10.3208	72.3466	103.3056
21	Apatity	0.0567	0.0270	0.0230	49.6539	57.0122	513.8190	16.1262	90.4332	129.1320
22	Kirovsk	0.0270	0.1630	0.1467	30.8957	35.4742	201.8820	5.3771	37.6926	53.8222
23	Revda	0.0624	0.0210	0.0080	54.7296	62.8401	407.2254	1.8311	23.7529	15.0139
24	Olenegorsk	0.7622	0.4800	0.0026	424.1424	303.1987	6156.8929	23.2447	248.3133	133.4122
25	Kovdor	0.0171	0.0754	0.0490	7.9781	179.4590	130.7440	34.4456	94.4123	133.2642
26	Kola	0.0097	0.0453	0.0294	4.7869	107.6754	89.5507	20.6673	56.6474	79.9585
27	Nikel	0.0116	0.0566	0.0368	5.9836	134.5942	22.3877	25.8342	70.8092	99.9482
28	Bilibino	0.0055	0.0368	0.0239	3.8893	87.4863	106.9571	16.7922	46.0260	64.9663

Source: Official Statistics of Russian Federal State Statistic Service [52]

Table S8. The input and output data of the Russian cities along the Northern Sea Route in 2017

No.	Russian city	Population (million people)	Capital (billion US dollars)	Public investment (billion US dollars)	Water supply (average daily consumption in thousand m^3)	Energy supply (specific fuel consumption for electric power generation by thermal power plants in grams of conventional	GRP (million US dollars)	GHG (thousand tons of CO_2 equivalent)	Solid waste (thousand tons)	Water pollution (million m^3)
1	St. Petersburg	5.2810	19.4300	9.4071	1555.9000	258.6000	54636.8286	169.3000	1926.0000	989.0000
2	Murmansk	0.2980	2.0634	0.3500	311.1800	300.0400	4004.3751	6.8800	209.1000	322.0000
3	Kandalaksha	0.0320	0.0202	0.0019	49.7888	48.0064	311.3400	1.3072	46.0020	57.9600
4	Onega	0.0190	0.0029	0.0008	5.6725	5.4186	202.3710	2.1126	6.6548	8.5540
5	Arkangelsk	0.3580	0.2057	0.0557	405.1800	387.0400	2819.7475	150.9000	475.3400	611.0000
6	Naryan Mar	0.0247	1.5229	3.5913	34.8522	33.6045	1903.4286	5.0224	152.6430	235.0600
7	Dudinka	0.0215	0.0580	0.0110	248.9440	240.0320	107.2300	1.0458	36.8016	46.3680
8	Providenya	0.0021	0.0000	0.0190	4.4725	4.2186	43.6389	1.1072	36.5720	49.4400
9	P.-Kamchatskiy	0.1805	0.5571	0.1614	234.0074	213.6285	1880.0000	7456.0000	177.7350	267.2600
10	Vanino	0.0155	0.0019	0.0520	40.8268	39.3652	146.1587	1.1111	46.0020	57.9600
11	Vladivostok	0.6066	1.7860	0.2275	345.7556	252.0336	6305.0000	18.8111	214.0000	109.8889
12	Hakhodka	0.1514	2.9100	0.0416	263.3670	328.9840	1703.5001	72.4320	228.1632	293.2800
13	Novodvinsk	0.0387	0.1500	0.0005	45.3078	43.6858	390.0000	6.5291	257.9667	305.5780
14	Vorkuta	0.0581	0.0800	0.0300	61.1655	58.9759	700.6206	2.0316	23.1120	11.8680
15	Salekhard	0.0485	0.2300	0.1035	42.8159	50.1295	3823.1800	1.4221	19.6452	11.8680

16	Nadym	0.0447	0.0000	0.0000	36.3935	42.6101	3592.4300	1.2088	16.6984	10.0878
17	N. Urengoy	0.1133	0.2706	0.0466	25.3689	276.3466	8213.0000	61.5672	193.9387	249.2880
18	Noyabrsk	0.1069	4.2400	2.7560	22.5783	240.4215	7653.0000	54.7948	168.7267	214.3877
19	Norilsk	0.1780	0.9000	0.5494	297.6047	371.7519	1663.6957	81.8482	257.8244	331.4064
20	Monchegorsk	0.0426	0.1000	0.0900	38.5343	45.1165	412.4330	9.8508	77.5755	99.7152
21	Apatity	0.0564	0.0310	0.0264	48.1679	56.3957	532.4330	15.3918	96.9694	124.6440
22	Kirovsk	0.0267	0.1520	0.1368	29.9711	35.0906	231.5520	5.1322	40.4168	51.9516
23	Revda	0.0626	0.0280	0.0106	53.0917	62.1606	438.3533	1.7634	24.3600	14.7163
24	Olenegorsk	0.7576	0.0190	0.0035	411.4491	299.9200	6768.4500	22.3852	254.6600	130.7678
25	Kovdor	0.0169	0.0624	0.0405	8.1282	177.4215	144.3962	32.8769	101.2360	128.6326
26	Kola	0.0097	0.0374	0.0243	4.8769	106.4529	98.9015	19.7261	60.7416	77.1796
27	Nikel	0.0116	0.0468	0.0304	6.0961	133.0661	24.7254	24.6577	75.9270	96.4745
28	Bilibino	0.0053	0.0304	0.0198	3.9625	86.4930	118.1255	16.0275	49.3526	62.7084

Source: Official Statistics of Russian Federal State Statistic Service [52]

Table S9. The input and output data of the Russian cities along the Northern Sea Route in 2018

No.	Russian city	Population (million people)	Capital (billion US dollars)	Public investment (billion US dollars)	Water supply (average daily consumption in thousand m^3)	Energy supply (specific fuel consumption for electric power generation by thermal power plants in grams of conventional	GRP (million US dollars)	GHG (thousand tons of CO_2 equivalent)	Solid waste (thousand tons)	Water pollution (million m^3)
1	St. Petersburg	5.3510	21.2100	10.6771	1540,3	258.1000	59907.0000	164.2000	1976.0000	963.0000

2	Murmansk	0.2950	2.4171	0.3300	302.3300	298.4500	4406.0006	7.3500	224.6000	301.0000
3	Kandalaksha	0.0310	0.0229	0.0004	48.3728	47.7520	321.4600	1.3965	49.4120	54.1800
4	Onega	0.0180	0.0031	0.0011	5.5486	5.3963	208.9490	2.0762	6.9230	8.3860
5	Arkangelsk	0.3590	0.2229	0.0800	396.3300	385.4500	3102.5587	148.3000	494.5000	599.0000
6	Naryan Mar	0.0248	1.3000	5.2379	33.8610	33.4264	2029.7143	5.3655	163.9580	219.7300
7	Dudinka	0.0210	0.0600	0.0110	241.8640	238.7600	108.6600	1.1172	39.5296	43.3440
8	Providenya	0.0022	0.0000	0.0121	4.3486	4.1963	46.9943	1.1965	39.9820	45.6600
9	P.-Kamchatskiy	0.1812	0.5610	0.1914	227.3522	212.4964	2138.5714	7933.0000	190.9100	249.8300
10	Vanino	0.0153	0.0027	0.0766	39.6657	39.1566	158.1911	1.1870	49.4120	54.1800
11	Vladivostok	0.6049	1.8800	0.2145	332.0740	250.6980	6685.7143	18.2444	219.5556	107.0000
12	Hakhodka	0.1493	3.1100	0.0444	257.6145	327.6325	2024.8005	71.1840	237.3600	287.5200
13	Novodvinsk	0.0383	0.1300	0.0001	44.0192	43.4543	409.5238	6.9752	277.0890	285.6490
14	Vorkuta	0.0561	0.0400	0.0090	59.4260	58.6633	1279.7295	1.9704	23.7120	11.5560
15	Salekhard	0.0492	0.5660	0.2547	41.5982	49.8638	4077.7310	1.3793	20.1552	11.5560
16	Nadym	0.0446	0.0000	0.0000	35.3585	42.3843	3768.6500	1.1724	17.1319	9.8226
17	N. Urengoy	0.1148	0.3143	0.1146	25.7235	275.2113	8732.4500	60.5064	201.7560	244.3920
18	Noyabrsk	0.1069	6.2000	4.0300	22.8939	239.4338	8172.4500	53.8507	175.5277	210.1771
19	Norilsk	0.1796	0.9971	0.5983	291.1044	370.2247	1776.6196	80.4379	268.2168	324.8976
20	Monchegorsk	0.0421	0.2100	0.1890	37.4384	44.8774	432.3210	9.6810	80.7024	97.7568
21	Apatity	0.0557	0.0380	0.0323	46.7980	56.0968	552.3210	15.1266	100.8780	122.1960
22	Kirovsk	0.0266	0.1830	0.1647	29.1187	34.9047	219.3450	5.0438	42.0460	50.9313
23	Revda	0.0627	0.0230	0.0087	51.5818	61.8312	415.0399	1.7103	24.9924	14.3294
24	Olenegorsk	0.7536	0.0200	0.0029	395.1681	298.3306	7107.2857	21.7109	261.2711	127.3300
25	Kovdor	0.0166	0.0912	0.0593	8.2418	176.4338	154.1972	32.3104	105.3166	126.1063
26	Kola	0.0097	0.0547	0.0356	4.9451	105.8603	105.6145	19.3863	63.1900	75.6638
27	Nikel	0.0114	0.0684	0.0444	6.1814	132.3254	26.4036	24.2328	78.9875	94.5797

28	Bilibino	0.0053	0.0444	0.0289	4.0179	86.0115	126.1433	15.7513	51.3419	61.4768

Source: Official Statistics of Russian Federal State Statistic Service [52]

Table S10. The input and output data of the Russian cities along the Northern Sea Route in 2019

No.	Russian city	Population (million people)	Capital (billion US dollars)	Public investment (billion US dollars)	Water supply (average daily consumption in thousand m^3)	Energy supply (specific fuel consumption for electric power generation by thermal power plants in grams of conventional	GRP (million US dollars)	GHG (thousand tons of CO_2 equivalent)	Solid waste (thousand tons)	Water pollution (million m^3)
1	St. Petersburg	5.3830	17.9100	11.3586	1489.7000	257.6000	55285.7100	149.1100	2026.0000	951.0000
2	Murmansk	0.2920	2.4429	0.2600	297.9400	297.3400	4868.4720	8.1500	243.7000	288.0000
3	Kandalaksha	0.0300	0.0265	0.0005	47.6704	47.5744	332.6500	1.5485	53.6140	51.8400
4	Onega	0.0180	0.0036	0.0015	5.4872	5.3808	216.2225	2.0454	7.2971	8.2740
5	Arkangelsk	0.3590	0.2543	0.1086	391.9400	384.3400	3374.2202	146.1000	521.2200	591.0000
6	Naryan Mar	0.0248	1.3671	7.3644	33.3693	33.3021	2167.4286	5.9495	177.9010	210.2400
7	Dudinka	0.0210	0.0680	0.0120	238.3520	237.8720	125.0000	1.2388	42.8912	41.4720
8	Providenya	0.0022	0.0000	0.0132	4.2872	4.1808	46.9888	1.3485	44.1840	43.3200
9	P.-Kamchatskiy	0.1812	0.5769	0.2043	224.0509	211.7061	2163.5714	7566.0000	207.1450	239.0400
10	Vanino	0.0153	0.0026	0.0720	39.0897	39.0110	158.1714	1.3162	53.6140	51.8400
11	Vladivostok	0.6050	7.5700	0.1690	331.0444	249.7656	6444.2857	16.5678	225.1111	105.6667
12	Hakhodka	0.1475	3.2500	0.0464	254.7610	326.6890	2333.4336	70.1280	250.1856	283.6800
13	Novodvinsk	0.0380	0.0700	0.0007	43.3801	43.2927	397.1429	7.7344	300.6527	273.3120

14	Vorkuta	0.0542	0.1200	0.0500	58.5631	58.4452	1483.6425	1.7893	24.3120	11.4120
15	Salekhard	0.0501	0.6400	0.2880	40.9942	49.6784	4456.5500	1.2525	20.6652	11.4120
16	Nadym	0.0447	0.0000	0.0000	34.8450	42.2266	3814.9100	1.0646	17.5654	9.7002
17	N. Urengoy	0.1169	0.3474	0.1296	26.1941	274.4188	9515.0657	59.6088	212.6578	241.1280
18	Noyabrsk	0.1061	7.3200	4.7580	23.3128	238.7443	8955.0657	53.0518	185.0123	207.3701
19	Norilsk	0.1810	1.0043	0.6026	287.8799	369.1586	1946.7534	79.2446	282.7097	320.5584
20	Monchegorsk	0.0415	1.0043	0.9039	36.8947	44.7105	458.4320	9.5374	85.0631	96.4512
21	Apatity	0.0552	0.0430	0.0366	46.1184	55.8882	578.4320	14.9022	106.3289	120.5640
22	Kirovsk	0.0262	0.2200	0.1980	28.6959	34.7749	262.5310	4.9690	44.3179	50.2511
23	Revda	0.0623	0.0300	0.0114	50.8328	61.6012	421.6407	1.5531	25.6248	14.1509
24	Olenegorsk	0.7481	0.0440	0.0038	393.9429	297.2211	6892.4143	19.7157	267.8822	125.7433
25	Kovdor	0.0164	0.1076	0.0700	8.3926	175.7443	168.9635	31.8311	111.0074	124.4220
26	Kola	0.0097	0.0646	0.0420	5.0356	105.4466	115.7284	19.0987	66.6044	74.6532
27	Nikel	0.0112	0.0807	0.0525	6.2944	131.8082	28.9321	23.8733	83.2555	93.3165
28	Bilibino	0.0053	0.0525	0.0341	4.0914	85.6754	138.2231	15.5177	54.1161	60.6557

Source: Official Statistics of Russian Federal State Statistic Service [52]
